# High Throughput ELISAs to Measure a Unique Glycan on Transferrin in Cerebrospinal Fluid: A Possible Extension toward Alzheimer's Disease Biomarker Development

**DOI:** 10.4061/2011/352787

**Published:** 2011-08-17

**Authors:** Keiro Shirotani, Satoshi Futakawa, Kiyomitsu Nara, Kyoka Hoshi, Toshie Saito, Yuriko Tohyama, Shinobu Kitazume, Tatsuhiko Yuasa, Masakazu Miyajima, Hajime Arai, Atsushi Kuno, Hisashi Narimatsu, Yasuhiro Hashimoto

**Affiliations:** ^1^Department of Biochemistry, Fukushima Medical University School of Medicine, 1 Hikarigaoka, Fukushima 960-1295, Japan; ^2^Disease Glycomics Team, RIKEN Advanced Science Institute, Wako 351-0198, Japan; ^3^Department of Neurology, Kamagaya General Hospital, Kamagaya 273-0100, Japan; ^4^Department of Neurosurgery, Juntendo University School of Medicine, 2-1-1 Hongo, Bunkyo-ku, Tokyo 113-8421, Japan; ^5^Research Center for Medical Glycoscience, National Institute of Advanced Industrial Science and Technology (AIST), Tsukuba 305-8568, Japan

## Abstract

We have established high-throughput lectin-antibody ELISAs to measure different glycans on transferrin (Tf) in cerebrospinal fluid (CSF) using lectins and an anti-transferrin antibody (TfAb). Lectin blot and precipitation analysis of CSF revealed that PVL (*Psathyrella velutina* lectin) bound an unique N-acetylglucosamine-terminated N-glycans on “CSF-type” Tf whereas SSA (*Sambucus sieboldiana* agglutinin) bound **α**2,6-N-acetylneuraminic acid-terminated N-glycans on “serum-type” Tf. PVL-TfAb ELISA of 0.5 **μ**L CSF samples detected “CSF-type” Tf but not “serum-type” Tf whereas SSA-TfAb ELISA detected “serum-type” Tf but not “CSF-type” Tf, demonstrating the specificity of the lectin-TfAb ELISAs. In idiopathic normal pressure hydrocephalus (iNPH), a senile dementia associated with ventriculomegaly, amounts of the SSA-reactive Tf were significantly higher than in non-iNPH patients, indicating that Tf glycan analysis by the high-throughput lectin-TfAb ELISAs could become practical diagnostic tools for iNPH. The lectin-antibody ELISAs of CSF proteins might be useful for diagnosis of the other neurological diseases.

## 1. Introduction

CSF (cerebrospinal fluid), which circulates within the ventricles of the brain and subarachnoid space, reflects the physiological and pathological conditions of the central nervous system [1]. In fact, CSF proteins are used as biomarkers to diagnose neurological diseases such as idiopathic normal pressure hydrocephalus (iNPH) [2–4] and Alzheimer's disease (AD) [5, 6] and may predict the disease onset in a preclinical stage [7]. Since iNPH and AD show similar phenotypes such as dementia and ventriculomegaly, it is difficult to distinguish the two diseases especially in elderly patients. Therefore, simultaneous measurements of a battery of iNPH biomarkers and AD biomarkers could help exact diagnosis of iNPH and AD.

We previously designated Tf-1 and Tf-2 as two isoforms of transferrin in CSF which are separable by SDS-PAGE and showed that the Tf-2/Tf-1 ratio is higher in iNPH patients than in non-iNPH patients [3, 4]. In that study we used immunoblotting method to detect Tf-1 and Tf-2, but the low throughput of immunoblotting makes it impractical for clinical use. Although sandwich ELISA (enzyme-linked immunosorbent assay) or latex photometric immunoassay is high throughput, Tf-1 and Tf-2 cannot be distinguished by these methods because Tf-1 and Tf-2 are different only in their glycan portion, and the antibodies against the protein portion of Tf cannot distinguish the two isoforms.

To establish a high throughput method to distinguish Tf-1 and Tf-2, we developed ELISAs using a combination of lectins and an anti-Tf antibody (TfAb). Tf-1 has a “CSF-type” biantennary asialo- and agalacto-complex type N-glycans with bisecting *β*1,4-N-acetylglucosamine (GlcNAc) and core *α*1,6-Fucose [3, 8], whereas Tf-2 in CSF has a “serum-type” biantennary N-glycans with *α*2,6-N-acetylneuraminic acid (NeuAc) [3, 9]. Because of their distinct terminal sugars, that is, GlcNAc on Tf-1 and *α*2,6-NeuAc on Tf-2, we chose PVL (*Psathyrella velutina *lectin) and SSA (*Sambucus sieboldiana* agglutinin) to detect Tf-1 and Tf-2 respectively. Our data showed that the lectin-TfAb ELISAs distinguish the two isoforms to be quantified and that the amounts of SSA-reactive Tf were higher in iNPH patients than in non-iNPH patients. Our newly established lectin-TfAb ELISAs are high throughput methods to measure “glycoforms” of transferrin which might be practical for clinical use. 

## 2. Materials and Methods

### 2.1. Patients

This study included 28 iNPH patients comprising 14 males and 14 females aged 75.2 ± 6.1 years (mean ± SD) and 18 non-iNPH patients comprising 10 males and 8 females aged 74.9 ± 5.2 years [3]. The iNPH patients were diagnosed using the clinical guidelines for iNPH issued by the Japanese Society of NPH [10]. A bolus infusion test and the tap test were performed routinely. Patients whose gait disturbance improves after the tap test, which removes 30 mL of CSF via a lumbar puncture, were treated with a shunt operation. Those who showed symptomatic improvement 1 month after the shunt operation were defined as iNPH patients while those who did not were defined as non-iNPH patients. In addition, those who did not show improvement after the tap test were classified as non-iNPH patients. The study was approved by the ethics committee of Fukushima Medical University (No. 613), which is guided by local policy, national law, and the World Medical Association Declaration of Helsinki.

### 2.2. Immunoblotting and Lectin Blotting

CSF samples were dissolved in Laemmli buffer, boiled for 3 min, and loaded onto SDS-polyacrylamide gels (SuperSep Ace; Wako Pure Chemical Industries, Osaka, Japan). After SDS-PAGE, the proteins were transferred to a nitrocellulose membrane (Bio-Rad Laboratories, Hercules, Calif, USA). The membrane was blocked in 3% skim milk, incubated sequentially with an anti-transferrin antibody (Bethyl Laboratories, Montgomery, Tex, USA) and a horseradish peroxidase-labeled anti-goat IgG (Jackson ImmunoResearch Laboratories, West Grove, Pa, USA), and developed using a Super Signal West Dura Extended Duration Substrate (Pierce Biotechnology, Rockford, Ill, USA). For lectin blotting, the transferred membrane was blocked in 1% BSA, incubated with a biotinylated PVL or biotinylated SSA (Seikagaku Corporation, Tokyo, Japan) followed by a horseradish peroxidase-labeled streptavidin (Takara, Shiga, Japan), and developed.

### 2.3. Lectin Precipitation

CSF was incubated with SSA-agarose (Seikagaku Corporation), and the bound proteins were precipitated by centrifugation. The unbound proteins were further incubated with PVL-agarose (Seikagaku Corporation), and the bound proteins were precipitated.

### 2.4. Purification of Tf-1

Tf-1 was purified from human CSF as described before [3]. Briefly, CSF was applied to a HiTrap Blue HP column (GE Healthcare, Buckinghamshire, UK). The unbound proteins were applied to a HiTrap Q HP column (GE Healthcare). The bound proteins were eluted with a linear gradient of NaCl from 0 to 300 mM. Tf-1 was eluted at 130 mM NaCl. Tf-1 was further purified by rechromatography with a HiTrap Q HP column. The concentration of the purified Tf-1 was determined by immunoblot analysis with commercially available human Tf (Sigma-Aldrich, St. Louis, Mo, USA) as the standard.

### 2.5. Lectin-Antibody ELISAs

For PVL-TfAb ELISA, a 96-well C8 Maxisorp Nunc immuno module plate (Nunc, Roskilde, Denmark) was coated with 2.5 *μ*g PVL (Seikagaku corporation) at 4°C overnight and blocked with 0.4% BlockAce (Dainippon Sumitomo Pharma, Osaka). Purified Tf-1 was used as the standard. The standards and CSF samples were appropriately diluted with PBST (phosphate-buffered saline/0.05% Tween-20), applied to the plate, and incubated at 4°C overnight. After three washes with PBST, the plate was incubated sequentially with anti-Tf antibody (Bethyl laboratories, Montgomery, Tex, USA) and horseradish peroxidase-labeled anti-goat IgG (Jackson ImmunoResearch Laboratories, West Grove, Pa, USA). After three washes with PBST, the wells were incubated with TMB solution (Wako, Osaka, Japan), and 1 N HCl was added to stop the reaction. Absorbances at 450 nm were measured by a plate reader (Bio-Rad Laboratories). CV (Coefficient of variation) of PVL-ELISA was 5.36%.

 For SSA-TfAb ELISA, anti-Tf antibodies (Cappel; ICN Pharmaceuticals, Aurora, Ohio, USA) were pretreated with 1.4 mM sodium periodate to abolish SSA epitopes on the antibody and coated on a 96-well plate. Human reference serum (Bethyl laboratories) containing 3 mg/mL serum Tf was used as the standard. The standards and CSF samples were appropriately diluted with TBS (Tris-buffered saline) containing 0.05% Tween 20 and 0.5 mM EDTA and applied to the plate and incubated at 4°C overnight. After three washes with TBST, the plate was incubated sequentially with a biotin-SSA (Seikagaku Corporation) and a horseradish peroxidase-labeled streptavidin (Takara). After three washes with TBST, the plate was incubated with the TMB substrate, and the absorbances at 450 nm were measured. CV of SSA-ELISA was 3.65%.

### 2.6. Statistical Analysis

Data were analyzed with SPSS version 17 (SPSS, Chicago, Ill, USA). Amounts of SSA-Tf and PVL-Tf were analyzed by the Student's *t*-test and Mann-Whitney *U* test, respectively.

## 3. Results

**Figure 1:**
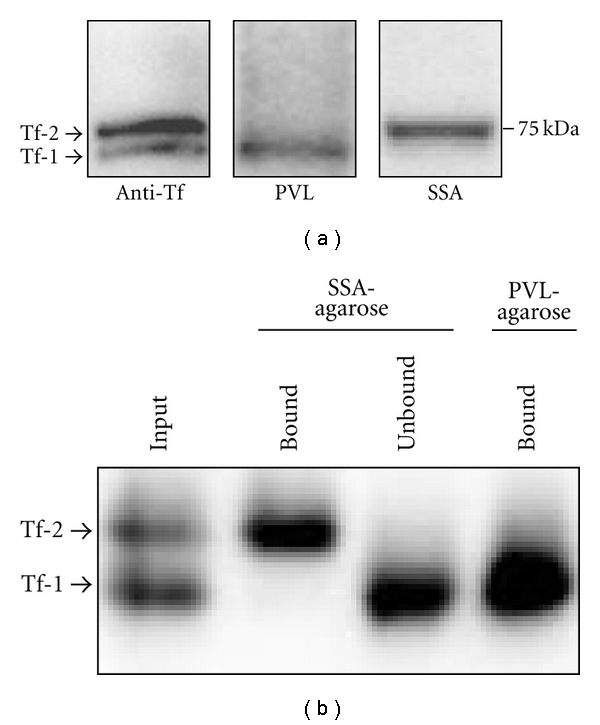
PVL and SSA specifically detect Tf-1 and Tf-2, respectively. (a) CSF was electrophoresed, blotted, and stained with anti-Tf antibody (left panel), PVL (center panel), and SSA (right panel). (b) CSF (input) was sequentially precipitated by SSA-agarose and PVL-agarose. The bound and unbound proteins were electrophoresed and immunoblotted by anti-Tf antibody.

To establish high throughput lectin-TfAb ELISAs that distinguish “CSF-type” Tf-1 and “serum-type” Tf-2, we first examined whether PVL and SSA specifically detect glycans on Tf-1 and Tf-2, respectively, by lectin-blotting. As we reported previously by immunoblotting, Tf-1 and Tf-2 in CSF were separated on SDS-gel (Figure 1(a) left). When PVL was used as a probe, a band with similar mobility to Tf-1 was detected in CSF (Figure 1(a) center), suggesting that PVL specifically detects the terminal GlcNAc on Tf-1 but not sugars on Tf-2. In contrast, when SSA was used as a probe, a band with similar mobility to Tf-2 was detected in CSF (Figure 1(a) right), suggesting that SSA, detects the terminal *α*2,6-NeuAc on Tf2 but not sugars on Tf-1. These band signals detected by PVL and SSA (Figure 1(a) center and right) were depleted by TfAb (data not shown), suggesting that the glycan epitopes detected by PVL and SSA reside on the Tf core protein. Moreover, when CSF was precipitated sequentially by SSA- and PVL-agarose, Tf-2 was specifically recognized by SSA, and Tf-1 was recognized by PVL (Figure 1(b)). Taken together, PVL and SSA can recognize Tf-1 and Tf-2, respectively, and distinguish the two Tf glyco-isoforms.

**Figure 2:**
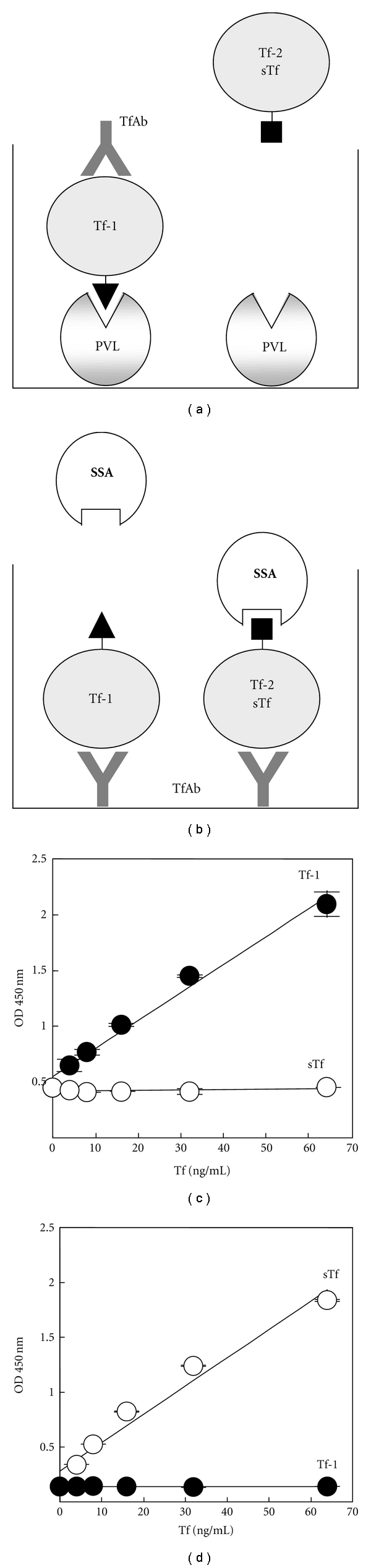
PVL-TfAb ELISA and SSA-TfAb ELISA specifically detect Tf-1 and Tf-2/serum Tf (sTf), respectively. (a) and (b). Schematic representation of lectin-TfAb ELISAs. PVL-TfAb ELISA (a) detects only Tf-1 while SSA-TfAb ELISA (b) detects only Tf-2/sTf. Closed triangles and rectangles represent “CSF-type” and “serum-type” glycans on Tf, respectively. (c) and (d) both the purified Tf-1 and serum Tf were measured in PVL-Tf ELISA (c) and SSA-TfAb ELISA (d). ODs at 450 nm were plotted at each concentration of each Tf. Closed and opened circles show the Tf-1 and serum Tf, respectively.

Next we investigated whether Tf-1 and Tf-2 were specifically detected by PVL-TfAb ELISA and SSA-TfAb ELISA systems, respectively (Figures 2(a) and 2(b)). The purified Tf-1 was used as a standard to measure Tf-1 in CSF by PVL-TfAb ELISA, whereas Tf in serum was used as a standard to measure Tf-2 by SSA-TfAb ELISA, since serum contains only “serum-type” Tf with very similar glycans to Tf-2 [3]. As shown in Figure 2(c), the purified Tf-1 was successfully detected in a dose-dependent manner (4–64 ng/mL) by PVL-TfAb ELISA, but no significant signals were detected with the serum Tf, indicating that the PVL-TfAb ELISA specifically detects Tf-1. Moreover, SSA-TfAb ELISA detected serum Tf (4–64 ng/mL) but not the purified Tf-1 (Figure 2(d)), suggesting specificity of the SSA-TfAb ELISA for serum Tf and Tf-2. Tfs which were detected by PVL-TfAb ELISA and SSA TfAb ELISA were designated as PVL-Tf and SSA-Tf, respectively.

**Figure 3:**
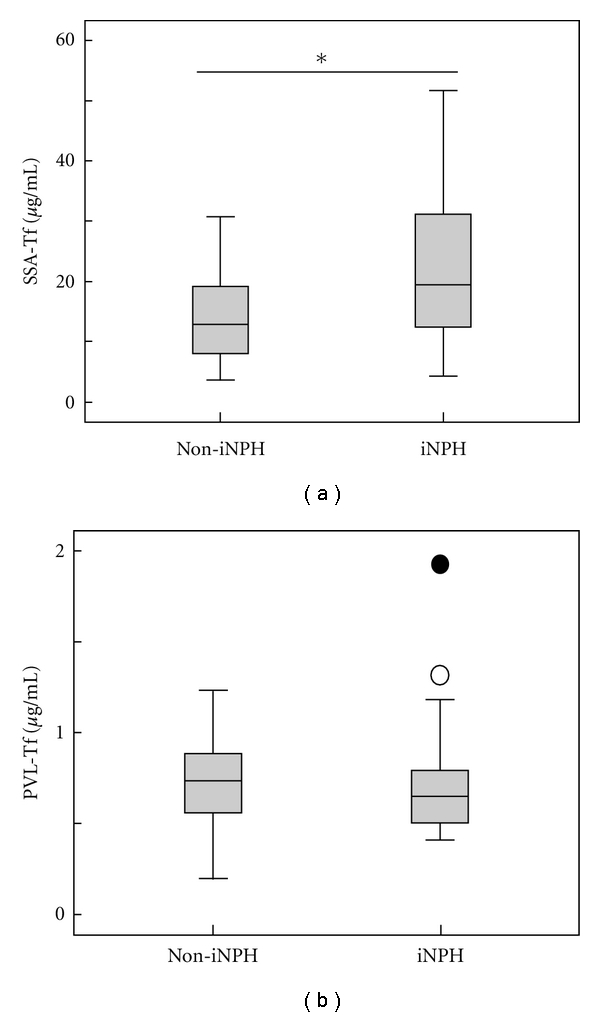
The SSA-Tf is increased in iNPH patients. Concentrations of SSA-Tf (a) and PVL-Tf (b) were measured in CSF of non-iNPH (*n* = 18) and iNPH (*n* = 28) patients, and box plots were shown. An asterisk indicates significantly different (*P* < 0.05). An open and closed circle represent an outlier and an extreme value, respectively.

Finally we measured the concentrations of PVL-Tf and SSA-Tf in CSF from iNPH and non-iNPH patients. As shown in Figure 3, amounts of SSA-Tf were significantly increased in iNPH patients compared to non-iNPH patients while amounts of PVL-Tf were not significantly different suggesting that the SSA-Tf might be an iNPH marker. The SSA-Tf/PVL-Tf ratio were also increased in iNPH patients (not shown), which is consistent with our previous data [3].

## 4. Discussion

In this study we have developed the high throughput lectin-TfAb ELISAs to measure Tf isoforms that have different terminal sugars. PVL-TfAb ELISA successfully detected Tf-1 but not Tf-2 whereas SSA-TfAb ELISA detected Tf-2 but not Tf-1, demonstrating that the lectin-TfAb ELISAs distinguish the two Tf isoforms to be quantified. Application of the method to iNPH patients revealed that amounts of SSA-Tf were significantly higher in patients with iNPH than in those without, suggesting that the lectin-TfAb ELISAs are promising high throughput methods for diagnosing iNPH. It would be interesting if the SSA-Tf or PVL-Tf is a diagnostic marker for AD or the Tfs can distinguish AD and iNPH.

The lectin-TfAb ELISAs have two differences compared to our previously developed immunoblotting method. First, the lectin-TfAb ELISA is more suitable for clinical use because the ELISA can process more samples. Second, the lectin-TfAb ELISAs detect amounts and structures of glycans on Tf while the immunoblot detects Tf core protein. The immunoblot is a well-established method for discovering biomarkers, but most glycoforms of CSF proteins, in contrast with Tf glycoforms, are not separable by SDS-PAGE (unpublished observation). In such cases, the lectin-antibody ELISAs are more useful to detect specific glycoforms than immunoblotting. Based on the concept that searches for biomarkers should involve not only “proteomics” but also “glycoproteomics” [11, 12], we are currently trying to develop ELISAs using various combinations of lectins and antibodies and to find new glycobiomarkers for iNPH as well as other neurological diseases such as AD.

## 5. Conclusion

We have developed the PVL-TfAb ELISA and SSA-TfAb ELISA to measure Tf-1 and Tf-2, respectively. Amounts of the SSA-reactive Tf were significantly higher in CSF of iNPH patients than in non-iNPH patients, suggesting that the lectin-TfAb ELISAs are promising high throughput methods for diagnosing iNPH. The lectin-antibody ELISAs might be useful for a CSF biomarker study of neurological diseases.
